# Quantification of Osseointegration of Plasma-Polymer Coated Titanium Alloyed Implants by means of Microcomputed Tomography versus Histomorphometry

**DOI:** 10.1155/2015/103137

**Published:** 2015-04-30

**Authors:** Carolin Gabler, Carmen Zietz, Richard Bieck, Rebecca Göhler, Tobias Lindner, Maximilian Haenle, Birgit Finke, Jürgen Meichsner, Holger Testrich, Mathias Nowottnick, Bernhard Frerich, Rainer Bader

**Affiliations:** ^1^Biomechanics and Implant Technology Research Laboratory, Department of Orthopaedics, University Medical Center Rostock, Doberaner Straße 142, 18057 Rostock, Germany; ^2^Leibniz Institute for Plasma Science and Technology (INP), Felix-Hausdorff-Straße 2, 17489 Greifswald, Germany; ^3^Institute of Physics, University of Greifswald, Felix-Hausdorff-Straße 2, 17489 Greifswald, Germany; ^4^Department of Informatics and Electrical Engineering, Institute of Device Systems and Circuit Technology, University of Rostock, Albert-Einstein-Straße 2, 18059 Rostock, Germany; ^5^Department of Oral and Maxillofacial Surgery, University Medical Center Rostock, Schillingallee 35, 18057 Rostock, Germany

## Abstract

A common method to derive both qualitative and quantitative data to evaluate osseointegration of implants is histomorphometry. The present study describes a new image reconstruction algorithm comparing the results of bone-to-implant contact (BIC) evaluated by means of *µ*CT with histomorphometry data. Custom-made conical titanium alloyed (Ti6Al4V) implants were inserted in the distal tibial bone of female Sprague-Dawley rats. Different surface configurations were examined: Ti6Al4V implants with plasma-polymerized allylamine (PPAAm) coating and plasma-polymerized ethylenediamine (PPEDA) coating as well as implants without surface coating. After six weeks postoperatively, tibiae were explanted and BIC was determined by *µ*CT (3D) and afterwards by histomorphometry (2D). In comparison to uncoated Ti6Al4V implants demonstrating low BIC of 32.4% (histomorphometry) and 51.3% (*µ*CT), PPAAm and PPEDA coated implants showed a nonsignificant increase in BIC (histomorphometry: 45.7% and 53.5% and *µ*CT: 51.8% and 62.0%, resp.). Mean BIC calculated by *µ*CT was higher for all surface configurations compared to BIC detected by histomorphometry. Overall, a high correlation coefficient of 0.70 (*p* < 0.002) was found between 3D and 2D quantification of BIC. The *μ*CT analysis seems to be suitable as a nondestructive and accurate 3D imaging method for the evaluation of the bone-implant interface.

## 1. Introduction

The surface characteristics of uncemented orthopaedic implants play an important role in the success or failure of implant anchoring after total joint replacement. For the long-term survival of uncemented implants, an appropriate ongrowth of bone cells is required [1]. Therefore, an important goal in endoprosthetic treatment implantology is a rapid bond and healing of the bone-implant interface. Osteoblast functions can be promoted by influencing the surface topography on the one hand [2, 3] and, on the other hand, cellular adhesion can be influenced by changing the surface chemistry [2, 4, 5]. For example, by means of plasma-polymerization, positively charged, nanometre-thin coatings can be deposited to the implant surfaces [6]. In particular, nitrogen-rich plasma-polymer coatings using the monomers allylamine and ethylenediamine with nitrogen functional groups seem to be suitable for the enhancement of adhesion and ongrowth of human osteoblasts [7, 8].

Therefore, the quantitative analysis of the response of bone tissue is of essential importance for the evaluation of the suitability of implants in terms of their design, the materials used, and surface modifications which aim at the improvement of osseointegration. Different analysis techniques to qualify and quantify the bone-to-implant interface have been developed. Histomorphometry is one of the most commonly used methods to qualify bone morphology and architecture and to quantify bone ongrowth. This method offers high resolution and image contrast and has been proven to be a reliable method to provide both qualitative and quantitative data in order to evaluate the osseointegration of implants. However, it is time and cost consuming due to the substantial preparation of specimens [9]. Further, this method presents risk adverse changes or damage to bone tissue during preparation and slicing [10]. The destructive nature of the procedure impedes the specimen from being used for other measurements and, therefore, this is one of its particular limitations. Additionally, the results are often based only on one or a few sections of the bone-implant interface. Histomorphometry provides only two-dimensional (2D) data and is based on the assumption that the analysis of the chosen sections is representative of the entire specimen [11, 12]. Hence, the optimal way to evaluate bone-implant contact is the subject of controversial discussion [13, 14].

To overcome these limitations, new imaging techniques and corresponding analysis methods are required. Microcomputed tomography (*μ*CT) is a promising technique to visualize and quantify three-dimensional (3D) structures, such as cortical and trabecular bone [15]. The major advantages, besides providing 3D data sets, are the reduced effort for sample preparation and, therefore, a shorter time to evaluation and the fact that this method is nondestructive. Nevertheless, the low image quality compared to histomorphometry of thin sections, for example, a poorer resolution and image artefacts in the presence of metallic implants, is the major disadvantage of *μ*CT. Therefore, the evaluation of bone-implant contact is the subject of controversial discussion [13, 14, 16]. In recent studies, trabecular bone with nonmetallic implantable devices was used to validate *μ*CT as an alternative technique for histomorphometry with a high correlation between both methods [16–19]. Certainly, the presence of metallic implants can lead to metallic halation and beam hardening [20–23]. However, the *μ*CT procedure for quantitative evaluation of bone structure has not been standardized so far.

The aim of the present study was to examine the suitability and validity of *μ*CT as a tool for the precise evaluation of bone-implant contact (BIC) in the case of titanium implant presence. Therefore, a new image reconstruction algorithm was described, and the results of BIC evaluated by means of the 3D data of the *μ*CT analysis were compared with the 2D results derived from histomorphometry.

## 2. Materials and Methods

### 2.1. Implant Material and Surface Coatings

Custom-made conical titanium alloyed (Ti6Al4V) implants providing a maximum outer diameter of 3 mm, a length of 3 mm, and a blasted surface using hydroxyapatite particles were used [24]. Three different surface configurations were examined: Ti6Al4V implants with plasma-polymerized allylamine (PPAAm) coating, those with plasma-polymerized ethylenediamine (PPEDA) coating, and those without specific surface coating as a reference.

The PPAAm coating was applied in a two-step procedure. First, the titanium sample was decontaminated and activated by continuous-wave oxygen plasma (500 W, 50 Pa, and 100 sccm O2/25 sccm Ar). The monomer allylamine was plasma-polymerized by the subsequent use of a pulsed (duty cycle of 0.15 at a pulse length of 2 s) microwave plasma source (2.45 GHz, 500 W) under low pressure (50 Pa) for an effective treatment duration of 144 s using argon-allylamine gas mixture [6].

For the PPEDA coating, a capacitive coupled radio frequency discharge (13.56 MHz) was used as plasma source. The metallic implant surface acted as electrode for the discharge. The implant coating was applied by use of an argon-ethylenediamine gas mixture, a total pressure of 60 Pa, and radio frequency forward power between 60 and 100 W [6, 25].

The thickness of tested PPAAm and PPEDA coatings was approximately 50 nm [6], thus the coatings did not change the surface topography. The adhesive bonding strength of the plasma-polymerised coatings was previously calculated according to the test standard DIN EN 582 [26]. There, coated cylindrical test samples made of titanium with 25 mm diameter were used. The adhesive bonding strengths of the coatings were higher than the 22 N/mm² required for medical implant surface coatings by ASTM standard 4711-F [27].

### 2.2. Animal Testing

The animal experiments were approved by the local review board of the Landesamt für Landwirtschaft, Lebensmittelsicherheit und Fischerei M-V (LALLF MV, reference number 7221.3-1.1-031/09). Surgery was performed on female Sprague-Dawley rats (Charles River Laboratories, Germany GmbH, Sulzfeld, Germany) with a body weight of 293 ± 23 g under general anaesthesia [24]. Using a circular drill (diameter: 2.8 mm), a bone defect was prepared bilaterally at the medial proximal tibiae, and the implants were subsequently inserted into the defects with press-fit [8, 24].

**Figure 1:**
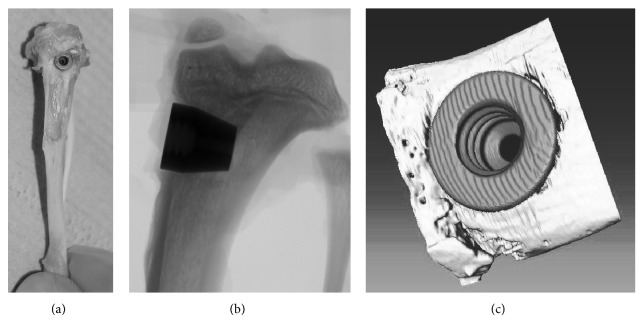
(a) Sample of rat tibia with implant freed from soft tissue. (b) Two-dimensional X-ray image of a tibia with implant. (c) Three-dimensional sectional view of a reconstructed volume of tibia with an inserted implant.

A total of 12 rats (*n* = 3 for experiments with uncoated implants; *n* = 4 for PPAAm coating; *n* = 5 for PPEDA coating) were observed for six weeks postoperatively. The animals were sacrificed and subsequently the tibiae were carefully dissected and freed of soft tissue (Figure 1(a)) and fixed in a buffered formalin solution (4%). A total of 15 implants (uncoated: *n* = 5; PPAAm: *n* = 4; PPEDA: *n* = 6) were used to determine bone-to-implant contact, first by *μ*CT and afterwards by histomorphometry. The remaining implants were used for microbiological investigations [24].

### 2.3. *μ*CT Analysis

Image data of the specimens were acquired individually with a microcomputer-tomograph (*μ*CT) Nanotom 180 nF (phoenix nanotom, GE Measurement & Control solutions, phoenix∣X-ray, Germany). For X-ray creation, a molybdenum target was used. Voltage and current were set to 70 kV and 135 *μ*A, to reach the optimum contrast. The used *μ*CT was a cone-beam computed tomograph with vertical specimen alignment. The samples rotated 360° in 0.75° steps. At each step, three 2D images were made (Figure 1(b)); altogether, 1,440 2D images were acquired from one sample. The region-of-interest (ROI) for the two-dimensional images was set in the range of the implant surface with approximately 1 mm surrounding bone tissue. Due to anatomical differences of the tibiae, the distance of the samples to the X-ray-tube and the detector, which consequently influences the magnification and voxel size, was varied such that the ROI was located inside the detector width with rotation of 360°. Voxel size and magnification of each sample are shown in Table 1. Each piece of the 2D data records includes a 7 GB data volume and was used for generation of the 3D volume of the sample (Figure 1(c)). The reconstruction of CT data (composing X-ray 2D images to a 3D volume) was performed with datos|X-reconstruction (GE, Germany). Titanium implants are harder to penetrate by X-rays in comparison to bone. Therefore, a beam hardening correction of 6.7 was used to compensate for the inhomogeneous reconstructed volume of the implant and reach similar grey values inside the entire implant. For further processing, the transformation of the volume data in the DICOM data was required.

### 2.4. Image Reconstruction

Each DICOM dataset had a maximum voxel edge resolution of 4-5 *μ*m and a data volume of roughly 5 GB. The segmentation algorithm was executed on a workstation (Intel Quad Core Q9400 2.66 GHz, 2 GB RAM) with the segmentation software Amira 5.4.1 (FEI Visualization Sciences Group, Oregon, USA) and consisted of the following processing steps: image data preprocessing, segmentation, and surface postprocessing.

Due to limited RAM capacity of the processing workstation, the data volume of each image set had to be resampled in order to reduce the amount of processed data. Datasets were resampled with a Box-filter (2x2x2-kernel), reducing data volume by factor 8 and voxel resolution by factor 2 while preserving spatial voxel proportions. Subsequently, datasets were filtered with an Unsharp-Masking algorithm (*x*-*y*-planes, 5*x*-kernels, sharpness 0.6) to improve the image quality for the following segmentation process. Additionally, datasets were cropped to reduce data volume and to approximate the volume of interest.

The segmentation algorithm comprised threshold detection of the histogram-oriented greyscale intensity of voxels followed by inspection and interactive editing of the segmented areas in each slice of a dataset.

**Figure 2:**
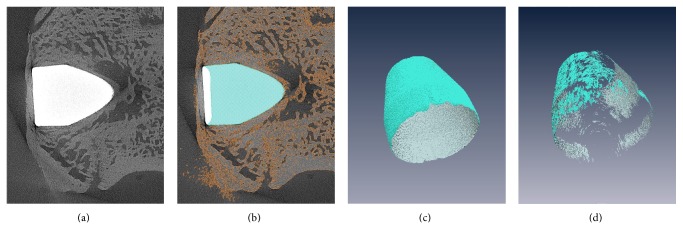
Steps of the data processing: (a) original sectional image from image dataset, (b) segmentation of bone and implant area in each sectional image, (c) reconstruction of the entire implant surface, and (d) image of the bone-to-implant contact area.

The possible bone-to-implant contact (pBIC) area was reduced to the shell area of the implants. For all slices, corrections at the transition from implant to bone were achieved with morphological opening and removing of islands. Errors occurring at the transition from bone to background areas due to image acquisition artefacts (black edges, structure blurring) were corrected manually. Following this, the segmentation surface models were computed with a voxel-sustaining algorithm matching the exact voxel boundaries of each segmented material. Since the surface information corresponds to the original number of triangles at the interface of two adjacent material surfaces, any sort of smoothing algorithm or operation had to be avoided. The interface patches of implant and bone materials were extracted from the reconstructed surface models. With the “Surface Area” module of Amira software, the exact number of triangles of the target patch was measured and converted into the corresponding real surface (Figure 2).

**Figure 3:**
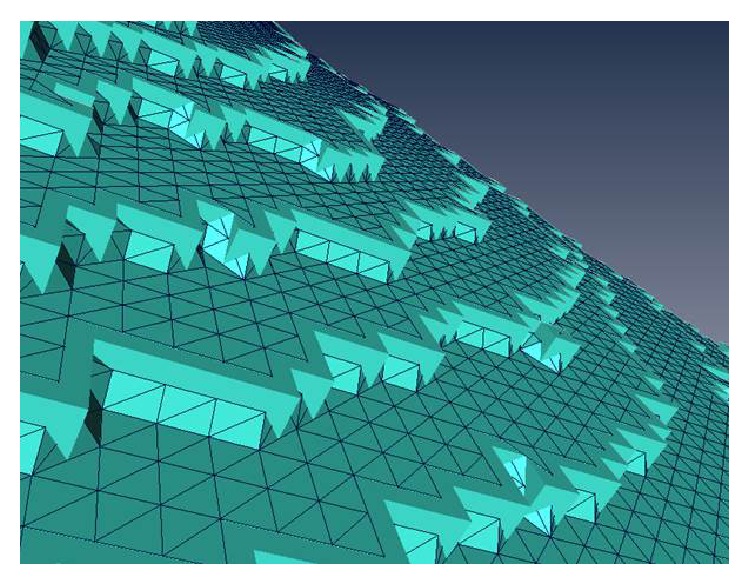
Image of the voxel-conserving surface calculation. The possible bone-to-implant contact (pBIC) area was calculated higher as implant shell area caused by Aliasing.

As a consequence of the voxel-conserving process, the possible bone-to-implant contact (pBIC) area and the effective bone-to-implant contact (eBIC) area were larger than the real implant shell area. This was assumed to be caused by Aliasing [28, 29] when a surface curvature is approximated by voxels (Figure 3). Therefore, a percentage value for the contact area (pVA) was calculated by dividing the eBIC by the pBIC.

For some surface models, the pBIC was reduced by adverse implant positioning during implantation. Implant shell areas were partially outside of the bone and had to be excluded from the segmentation. The exclusion was achieved by cropping the corresponding shell areas in the surface models. The cropping was executed in Geomagic (version 2012, Morrisville, NC, USA) with the “Plane Intersection” module. This step automatically resulted in a reduction of the pBIC and had to be corrected in the calculation of the pVA.

### 2.5. Histomorphometric Analysis

**Figure 4:**
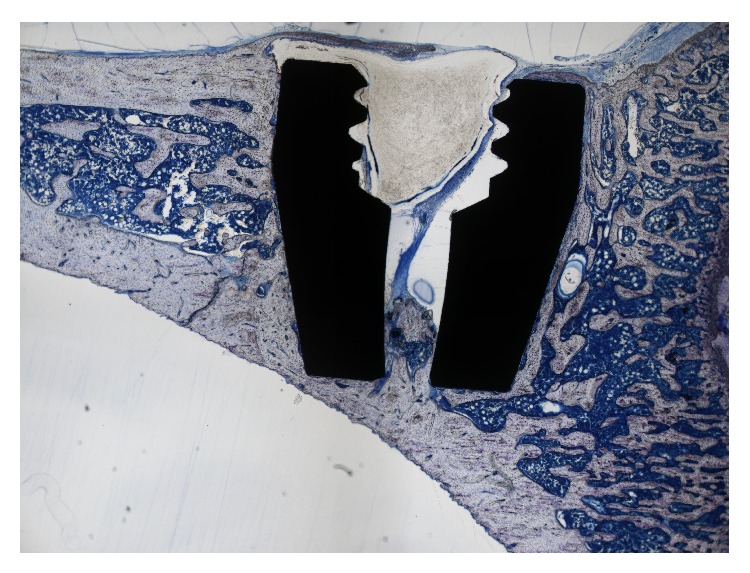
Histomorphometric sample with a PPAAm-coated implant in a proximal rat tibia (toluidine blue staining).

The specimens were dehydrated in a graded series of alcohol and embedded in polymethylmethacrylate as described by Kopp et al. [12]. For each implant, one slice in the longitudinal direction of the implant was prepared. The undecalcified bone sections were produced using a thin-section technique according to Donath and Breuner [30]. Sections were produced with cutting/grinding system with 0.1 mm diamond bands. The thickness of the sections was reduced to approximately 20 *μ*m using 1200-grit sandpaper and subsequently polished using 4000-grit polishing paper in a microgrinding system (Exact 400CS; Exakt Apparatebau) [12]. Finally the bone sections were stained with toluidine blue (Figure 4).

Histological evaluations were performed using a light microscope (Eclipse TS 100; Nikon, Japan, ×20 magnification) equipped with a digital camera (Nikon Digitale Sight DS-2 mV) and connected to a computer. The histomorphometric data were analysed by the software for microscopic images, NIS-Elements D 3.2 (Nikon Instruments Inc., Melville, NY, USA).

Referring the *μ*CT analysis, only the length of the shell area of the implants was evaluated. Only those parts of the implant were considered that were completely surrounded by bone tissue. Bone-to-implant contact (BIC) was determined as a percentage from the surface length overgrown with bone tissue to the total surface contact length of the implant.

### 2.6. Statistical Analysis

The statistical analysis was performed using SPSS Statistics 20 (IBM, Armonk, NY, USA). The statistical data includes mean and standard deviations. All values were proven to have homogeneity of variance (Levene test) before pair-wise comparisons within the independent groups were performed. The Mann-Whitney *U* test was used to compare the values of the groups because of the low number of the samples. Values of *p* < 0.05 were considered to be statistically significant.

## 3. Results

During the six-week implantation period, no signs of implant-associated infection or alterations in animal behaviour were observed. All plasma-coated and uncoated titanium implants showed direct bone contact in *μ*CT and histological analysis. However, there were differences in the extent of bone-to-implant contact (BIC) among the types of coating and the kinds of evaluation. The values are given in Table 2.

After six weeks of implant endurance in the tibia, the uncoated titanium implants demonstrated the lowest BIC in *μ*CT and histomorphometry evaluation. The plasma-polymerized coatings resulted in an increase of BIC. Compared to the uncoated implants, the PPAAm-coated implants showed a slight, nonsignificant increase in BIC in *μ*CT (*p* = 0.905) and in histomorphometry (*p* = 0.730). Implants with the PPEDA coating revealed a clear but not significant increase in BIC evaluated by *μ*CT (*p* = 0.329) and histomorphometry (*p* = 0.126). Between PPAAm and PPEDA, no significant difference (*μ*CT: *p* = 0.257, histomorphometry: *p* = 0.762) was found.

Mean BIC calculated by *μ*CT was higher for all surface modifications compared to BIC detected by histomorphometry, but the difference was not significant (*p* = 0.202). Furthermore, there was an obvious decrease in standard deviation (SD) when BIC was evaluated by *μ*CT. Overall, a high correlation coefficient of 0.70 (*p* < 0.002) was found between 3D and 2D quantification of BIC.

## 4. Discussion

Quantitative determination of the response of surrounding bone tissue is essential to assess the suitability of orthopaedic implants in terms of their design, surface modification, and the materials used, all of which aim at strengthening the bone response. Although 2D histomorphometry is time-consuming, destructive, and cost-intensive and the results are based only on one or a few implant sections, it is still one of the most commonly conducted methods to qualify bone morphology and to quantify the osseointegration of implants. Compared to histological evaluation, *μ*CT imaging is fast and nondestructive and offers 3D data sets. Therefore, this technology has become of increasing interest in recent years. The evaluation of trabecular bone by *μ*CT was validated as an alternative technique with a high correlation between *μ*CT and histology based morphometry. Müller et al. [17] presented quantitative morphometric analysis of human bone biopsies with correlations up to *r* = 0.93 and low differences, from 2.5 to 6.1%, between *μ*CT and histomorphometry. Similarly high correlations of bone volume determined due to histomorphometry and *μ*CT were approved by Chappard et al. (*r* = 0.93) [18] and Park (*r* = 0.85) [19]. The presence of metallic implants can lead to artefacts, such as metallic halation and beam hardening [20–23]. Therefore, evaluation methods for bone-implant contact characterisation are the subject of controversial discussion [13, 14, 16].

In the present study, the evaluation of BIC by means of *μ*CT was compared with the results evaluated by 2D histomorphometry. An overall significantly high correlation between *μ*CT and histomorphometry was found. The results demonstrate a nearly 11% higher mean BIC with decreased standard deviation calculated by *μ*CT as compared to BIC detected by histomorphometry. However, the differences were not significant.

Furthermore, it is difficult to compare our presented data with other studies due to different evaluated parameters and the variability of specimens, their dimensions, the presence of metallic implants, and the design of the implants. Higher BIC values determined by *μ*CT compared to histomorphometry have been described in other studies [13, 18, 31]. An overestimation of bone volume due to *μ*CT was shown in analysis with [13] and without [18] implants and was explained by the definition of the threshold procedure for *μ*CT analysis. The differences of BIC evaluated by histomorphometry and *μ*CT were also discussed; for example, Sarve et al. [31] gained 21% less BIC in 2D quantification. They compared their results with those of Rebaudi et al. [32], who presented about 20% greater bone-implant apposition obtained with 2D histomorphometry compared to *μ*CT and assumed that the slice thickness of 50 *μ*m of the histomorphometric specimens, like Rebaudi et al. used, resulted in overestimation of BIC, as described by Johansson and Morberg [33]. Schouten et al. [13] observed a thin layer of noise around the entire implant, which impeded the detection of BIC, although the bone trabeculae were clearly visible on the *μ*CT images. This led to BIC values, which were 5 to 30% higher compared to the histomorphometry values. Bernhardt et al. [34] were able to achieve relatively low deviations between the quantification of BIC with histomorphometry and the *μ*CT method. BIC for histomorphometry was slightly but significantly higher compared to that for *μ*CT (2.4%; *p* = 0.014). Surprisingly, they obtained a high correlation between histomorphometry and 2D *μ*CT (*r* = 0.968), but they found only a minor correlation between histomorphometry and 3D *μ*CT (*r* = 0.5). Another explanation for the overestimation of BIC due to *μ*CT is the lower resolution (voxel sizes 4.00 to 4.75 *μ*m) compared to histomorphometry (pixel size 1.10 *μ*m). A further reduction of the image resolution may result in a further overestimation of BIC. For example radiograms of such a small implant, as we used in our study, would falsely indicate a BIC of nearly 100%. Compared to other studies, which used voxel sizes of about 10 *μ*m [35–37] up to 20 *μ*m and higher [13, 38, 39], our *μ*CT analysis provides a proper voxel resolution.

Limited picture quality due to beam hardening artefacts at the margin of the implant led to a loss of information and impeded the image reconstruction. Therefore, separation of less dense bone structures and background areas was difficult. Beam hardening artefacts resulted from the use of polychromatic X-ray spectra and the inhomogeneous sample (bone with implant) [20]. Hence, beam hardening correction was used to compensate for the inhomogeneous reconstructed volume of the implants. For further evaluations, beam filtration, for example, with aluminium or copper, can be used to minimize these artefacts [40].

Due to limited RAM capacity, the data volume of each set had to be resampled in order to reduce the amount of processed data. The used Box-filter with twofold resampling resulted in a moderate reduction of the storage requirements at sufficiently well recognizable interface details and acceptable resolution. However, even with the use of this filter, the disadvantage factor contributing to the processing time of the data sets was up to 12 hours. Nevertheless, reduction of the storage requirements due to resampling and, therefore, a reduced voxel resolution involves the risk of negative influence on the evaluated effective BIC. Unsharp-Masking filtering in Amira software was used to improve data visualisation of relevant image structures for the following segmentation process, while achieving sufficient noise suppression and minimal loss of voxel information. The algorithm (5x-kernels, sharpness 0.6) offered a compromise between noise rejection and detail preservation. As a result, the darkest bone constituents were barely visible, but a good sharpening was still achieved. The filter has in large kernels (from 7 × 7) high-pass character, which is associated with the loss of voxel value lower intensities and could potentially lead to a loss of information in the bone area.

The implants used in the present study were designed to evaluate osseointegration and implant-associated infections in the tibial metaphysis of rats [8, 24]. Due to the implant design and the implantation procedure, the shell area of the implant had direct bone contact, and regenerated bone tissue had to fill adjacent defects postoperatively. Therefore, the BIC was only analysed at the shell area. Other implant designs, such as threaded dental implants with high primary stability, might be better suited for osseointegration tests [41]. On the other hand, such implants can lead to higher artefacts in the region of threads, resulting in additional effort for image processing as described by Schouten et al. [13].

Our present animal study was further limited by a low sample size for the comparative evaluation of implant osseointegration. We examined 15 implants by means of both *μ*CT and histomorphometry. In the histomorphometrical evaluation, only one slice per implant could be used to determine the BIC. The selection of the section plane is an important and influential factor in histomorphometric evaluations [12, 34, 37]. We assume that the low sample size could be one of the main reasons why significant differences between the surface modifications were not found in contrast to previous evaluations [8].

In further studies, imaging evaluation by *μ*CT can be extended by biomechanical tests, for example, push-out/pull-out or torque removal tests. It has been shown that significant ongrowth differences between implant modifications or evaluation time points found out by *μ*CT can be correlated to biomechanical evaluations [35, 38]. Stadlinger et al. [36] correlated the results of different parameters of osseointegration of biochemically modified implants. After an implantation period of four weeks, they recorded BICs by 3D *μ*CT and 2D histomorphometry with a high correlation value of 0.807 (*p* ≤ 0.01). BIC (*μ*CT), and removal torque resulted in a moderate correlation value of 0.614 (*p* ≤ 0.01), considering that the experimental group was divided for imaging analysis, where specimens were fixed in ethanol, and for biomechanical evaluation with fresh specimens. For further investigations, it is contemplated to examine additional parameters other than solely BIC due to histomorphometry and *μ*CT.

## 5. Conclusion

We found a high correlation between 3D *μ*CT and 2D conventional histomorphometric quantification of BIC. The use of *μ*CT is a nondestructive and precise procedure to gain 3D imaging data of the entire bone-implant interface. Furthermore, there was an obvious decrease in standard deviation when BIC was evaluated by *μ*CT. With the image reconstruction algorithm presented in this paper, limitations in the case of metallic specimens (streak artefacts) could be considerably reduced, and *μ*CT can be classified as a valuable technique to evaluate and quantify the osseointegration of titanium implants.

## Figures and Tables

**Table 1:** Magnification and voxel size of the samples.

Surface treatment	Sample number	Magnification	Voxel size [*μ*m]
Uncoated	1.1	11.50	4.35
	1.2	12.50	4.00
	1.3	12.00	4.17
	1.4	10.75	4.65
	1.5	11.22	4.46

PPAAm	2.1	11.36	4.40
	2.2	12.50	4.00
	2.3	11.50	4.35
	2.4	11.50	4.35

PPEDA	3.1	11.50	4.35
	3.2	11.75	4.26
	3.3	11.50	4.35
	3.4	11.50	4.35
	3.5	11.50	4.35
	3.6	10.53	4.75

**Table 2:** Bone-to-implant contact (mean ± standard deviation) evaluated by means of histomorphometry and *μ*CT.

Surface treatment	*N*	BIC ± SD (%) Histomorphometry	BIC ± SD (%) *μ*CT
Uncoated	5	32.4 ± 27.9	51.3 ± 11.6
PPEDA	6	53.5 ± 19.2	62.0 ± 9.6
PPAAm	4	45.7 ± 22.9	51.8 ± 13.3
Overall	15	44.4 ± 23.5	55.7 ± 11.7
